# Organizational factors associated with adherence to low tidal volume ventilation: a secondary analysis of the CHECKLIST-ICU database

**DOI:** 10.1186/s13613-020-00687-3

**Published:** 2020-06-01

**Authors:** Thais Dias Midega, Fernando A. Bozza, Flávia Ribeiro Machado, Helio Penna Guimarães, Jorge I. Salluh, Antonio Paulo Nassar, Karina Normílio-Silva, Marcus J. Schultz, Alexandre Biasi Cavalcanti, Ary Serpa Neto, for the CHECKLIST-ICU Investigators and the Brazilian Research in Intensive Care Network (BRICNet), Pedro Aniceto Nunes Neto, Carlos Alberto Fernandes, Cristiano Ávila Luchesi, Natalia Rodrigues de Sa, Roberta Antunes Faria Rodrigues, Larissa Stefany Radespiel, Vinicius Leonardo dos Santos Iorio, Danielle Bhering, Viviane Ribeiro Linhares, Glauco Adrieno Westphal, Geonice Sperotto, Soraya Byana Rezende da Silva Rossi, Thaismari Escarmanhani Ferreira, Edgar de Brito Sobrinho, Helder Jose Lima Reis, Mauricio Soares Carneiro, Adriana de Oliveira Lameira Veríssimo, Juliana Fernandez Fernandes, Rodrigo Lopes Ferreira, Sylvania Campos Pinho, Leonardo Dultra, Lise Oliveira Hizumi, Neiva Fernandes de Lima, Alexsandra Raimunda da Silva, Liliane dos Santos, Sidiner Mesquita Vaz, José Marcio Oliveira, Miria Bonjour Laviola, Wania Vasconcelos de Freitas, Leonardo Passos, Ligia Sarmet Cunha Farah Rabello, Carollina Resende de Siqueira, Luiz Carlos de Oliveira Silva, Francisco Felix Barreto Junior, Eduarda Maria Alves Cruz, Luiz Marcelo Sa Malbouisson, Fabiola Prior Caltabeloti, Estevão Bassi, Patrícia Regiane da Silva, Filipe Cadamuro, Renata Graciliano dos Santos Cagnon, Yeh-Li Ho, Lucas Chaves Neto, Bruno Azevedo Randi, Michele Maria Goncalves de Godoy, Pollyanna Dutra Sobral, Evônio de Barros Campelo Júnior, Laercio Martins de Stefano, Ana Lucia Gut, Greicy Mara Mengue Feniman de Stefano, Denise Milioli Ferreira, Fernanda Alves Ferreira Gonçalves, Fernanda Rubia Negrao Alves, Wilson Nogueira Filho, Renata Ortiz Marchetti, Sheila Sa, Nadia Tomiko Anabuki, Silvia Regina Rios Vieira, Edino Parolo, Karen Prado, Natalia Gomes Lisboa, Simone Castelo Branco Fortaleza, Maria Liduina Nantua Beserra Porfirio, Marcela Maria Sousa Colares, Rosicley Souza da Silva, Marcia Vasconcelos, Fabio de Souza, Lanese Medeiros de Figueiredo, Niedila Pinheiro Bastos Seabra, Paula Celia Pires de Oliveira, Marcio Andrade Martins, Ricardo Beduschi Muller, Manoela Cristina Recalcatti, Riani Helenditi Fernandes Camurça Martins, Leatrice Emilia Ferreira de França, Frederico Bruzzi de Carvalho, Gustavo Cesar Augusto Moreira, Evelin Drociunas Pacheco Cechinatti, Aline Cristina Passos, Mariana da Costa Ferreira, Antonio Carlos Babo Rodrigues, Vladimir dos Santos Begni, Alfredo Maximo Grilo Jardim, Amanda Carvalho Maciel, Marlus Muri Thompson, Erica Palacio Berçacola Pinheiro, Claudio Henrique Pinto Gonçalves, Ricardo Schilling Rosenfeld, Valéria Abrahao Rosenfeld, Leticia Japiassú, Maria Inês Pinto de Oliveira Bissoli, Rosemary da Costa Tavares, Marta Rocha Gonçalves, Laercia Ferreira Martins, Maria Helena Oliveira, Adriana Kelly Almeida Ferreira, Emídio Jorge Santos Lima, Tárcio de Almeida Oliveira, Milena Teixeira Campos, Nilce Almino de Freitas, Stephanie Wilkes da Silva, Vera Lucia Bento Ferreira, Alexander de Oliveira Sodré, Cid Leite Villela, Eduardo Duque Estrada Medeiros, Nairo Jose de Souza Junior, Jhocrenilcy de Souza Maya Nunes, Rones de Souza Monteiro, André Scazufka Ribeiro, Carlos Cesar Nogueira Giovanini, Elisete Tavares Carvalho, Lenise Castelo Branco Camurça Fernandes, Domitilha Maria Coelho Rocha, Cristiane Maria Gadelha de Freitas, Marcia Adélia de Magalhaes Menezes, Rosa Imaculada Stancato, Guilherme Brenande Alves Faria, Giovanna Asturi, Ricardo Reinaldo Bergo, Frederico Toledo Campo Dall’Orto, Gislayne Rogante Ribeiro, Cidamaiá Aparecida Arantes, Michelle Aparecida dos Santos Toneto, Cassiano Teixeira, Juçara Gasparetto Maccari, Sergio Tadeu Górios, Julliana Pires de Morais, Daniela Marangoni Zambelli, Roberta Machado de Souza, Eduardo Cenísio Teixeira de Paiva, Alessandra Gonçalves Ribeiro, Marcus Vinicius Pereira, Leoni Nascimento, Rosangela da Silva, Roberto de Almeida, Karin Aline Zilli Couto, Izabella Moroni Toffolo, Jorge Eduardo da Rocha Paranhos, Antonio Ricardo Paixão Fraga, Alberto Augusto de Oliveira Junior, Roberto Lannes, Andrea da Silva Gomes Ludovico, Luiz Fernando Costa Carvalho, Leticia de Araújo Campos, Patricia Soboslai, Israel Silva Maia, Tatiana Rassele, Christiany Zanzi, Valeria Nunes Martins Michel, Sinésio Pontes Gonçalves, Ricardo Rath de Oliveira Gargioni, Rosangela Zen Duarte, Mariza D’Agostino Dias, Andrea Delfini Diziola, Daniela Veruska da Silva, Melissa Sayuri Hagihara, André Luís Veiga de Oliveira, Diego Leonnardo Reis, Janaina Feijó, Marco Antonio da Costa Oliveira, Luiza Veiga Coelho de Souza, Liane de Oliveira Cavalcante, Jacilda Rodrigues, Moises Cruz de Pinho, Marcos Antonio Gadelha Maia, Vladia Fabiola Jorge Lima, Emilianny Maria Nogueira, Antonielle Carneiro Gomes, Katia Regina de Oliveira, Jose Antonio Bandeira, Carla Cordeiro Botelho Mesquita, Guilherme Arcaro, Camila Wolff, Délcio Caran Bertucci Filho, Gustavo Navarro Betonico, Rafaela Pereira Maroto, Leonardo Fantinato Menegon, Patrício Junior Henrique da Silveira, Germana Estrela Gadelha de Queiroga Oliveira, Wallber Moreno da Silva Lima, Lilian Batista Nunes, Sotero Gonçalves Martins Neto, Liwcy Keller de Oliveira Lopes Lima, Maria Augusta de Mendonça Lima, Lidiane Miranda Milagres, Vivian Gribel D’Ávila, Paulo Marcelo Schiavetto, Paulo Alves dos Santos, Francislaine de Matos Raimundo, Carmen Leonilia Tavares de Melo, Aline Albuquerque de Carvalho, Cynthia Franca de Santana, Adriana Lessa Ventura Fonseca, Martha Aparecida da Silva, Mara Lilian Soares Nasrala, Eloisa Kohl Pinheiro, Mara Regina Pereira Santos, Guilherme Abdalla da Silva, Rener Moreira, Marcia Loureiro Sebold, Marcone Lisboa Simões da Rocha, Marco Antonio Ribeiro Leão, Jaqueline de Assis, Felipe Dal Pizzol, Cristiane Damiani Tomasi, Jose Nivon da Silva, Luciana Vladia Carvalhedo Fragoso, Denise Araújo Silva Nepomuceno Barros, Mauricio Mattos Coutinho, Robson Sobreira Pereira, Veronica Oliveira dos Santos, Sergio Kiffer Macedo, Diego de Souza Bouzaga Furlani, Eduardo Silva Aglio Junior, Aécio Flavio Teixeira de Góis, Kathia Teixeira, Paula Zhao Xiao Ping, Paulo Ricardo Nazario Viecili, Simone Daniela Melo de Almeida, Geovani Moreno Santos Junior, Djalma Novaes Araújo Segundo, Marielle Xavier Santana, Jose Oliveira Calvete, Luciana Renner, Vinicius Vandré Trindade Francisco, Danytieli Silva de Carvalho, Adriana Neri da Silva Batista Campos, Sergio Leôncio Fernandes Curvelo, Nayara Ribas de Oliveira, Suelem de Paula Freitas Deborssan, Maria Raquel dos Anjos Silva Guimaraes, Luiz Claudio Gomes Bastos, Luciene Moraes Gomes, Erico de Lima Vale, Dionísia Arianne Vieira da Silva, Renato Vieira Gomes, Marco Antonio Mattos, Pedro Miguel Matos Nogueira, Viviane Cristina Caetano Nascimento, Cintia Magalhaes Carvalho Grion, Alexsandro Oliveira Dias, Glaucia de Souza Omori Maier, Paula Frizera Vassallo, Maria Helena Buarque Souza de Lima, Wyllyam Loss dos Reis, Walace Lirio Loureiro, Andressa Tomazini Borghardt, Ciro Leite Mendes, Paulo Cesar Gottardo, Jose Melquiades Ramalho Neto, Eliane Pereira da Silva, Maria Gorette Lourenço da Silva Aragao, Elisângela Maria de Lima, Rafael Lisboa de Souza, Ken Sekine Takashiba, Dimitri Gusmão-Flores, Taciana Lago Araújo, Rosana Santos Mota, Almir Germano, Flavia Antunes, Sandra Regina Bin Silva, Marcio Osório Guerreiro, Marina Peres Bainy, Patrícia de Azevedo Duarte Hardt, Hugo Correa de Andrade Urbano, Camila Amurim de Souza, Claudia Mangini, Fernando Jose da Silva Ramos, Luany Pereira de Araújo, André Miguel Japiassú, Denise Machado Medeiros, Michele Fernanda Borges da Silva, Fabiano Hirata, Roberto Marco, Elzo Peixoto, Marcio Luiz Fortuna Esmeraldo, Leide Aparecida Damásio Pereira, Raquel Carvalho Leal, Paulo Roberto Aranha Torres, Maricy Morbin Torres, Mara Rubia de Moura, Claudio Dornas de Oliveira, Andressa Siuves Gonçalves Moreira, Brisa Emanuelle Silva Ferreira, Carolina Leticia dos Santos Cruz, Patrícia Moreira Soares, Paulo Cesar Correia, Lorena Lina Silva Almeida, Marcelo Ferreira Sousa, Andrey Antonio Santiago Vial, Marcia Maria Ferreira Souza, Diogo Quintana, Ana Cecilia Barbosa Guimaraes, Maria Dolores Menezes Diniz, Paulo Henrique Panelli Ferreira, Rosa Maria Rios Santana Cordeiro, Murilo Oliveira da Cunha Mendes, Sergio Oliveira de Lima, Silvia Aparecida Bezerra, Aurélia Baquiao dos Reis da Silva, Cristiano Correa Batista, Thaís Neumann, Rafael Olivé Leite, Thiago Costa Lisboa, Martha Hädrich, Edison Moraes Rodrigues Filho, Jorge Luiz da Rocha Paranhos, Carlos Henrique Nascimento dos Santos, Hélia Cristina de Souza, Robson Viera de Souza, Jose Luís Toribio Cuadra, Jonas Spanholi, Marcos de Carvalho Borges, Wilson Jose Lovato, Tania Mara Gomes, Luís Artur Mauro Witzel Machado, Thiago Martins Santos, Marco Antonio de Carvalho Filho, Karina Aparecida Garcia Bernardes, Jose Roberto Pereira Santos, Aline Esteves Mautoni Queiroga Liparizi, Patrícia Venturim Lana, Claudio Piras, Luiz Virgílio Nespoli, Aparecida Silva Taliule

**Affiliations:** 1 Department of Critical Care Medicine, Hospital Israelita Albert Einstein, Albert Einstein Avenue, 700, São Paulo, Brazil; 2 Research Institute, Instituto D’Or de Pesquisa e Ensino (IDOR), Rio de Janeiro, Brazil; 3 Instituto Nacional de Infectologia Evandro Chagas, Fundação Oswaldo Cruz (FIOCRUZ), Rio de Janeiro, Brazil; 4 Anesthesiology, Pain and Intensive Care Department, Federal University of São Paulo, São Paulo, Brazil; 5 Academic Research Organization, Hospital Israelita Albert Einstein, São Paulo, Brazil; 6 Graduate Program in Translational Medicine and Department of Critical Care, Instituto D’Or de Pesquisa e Ensino (IDOR), Rio de Janeiro, Brazil; 7 Post Graduate Program in Internal Medicine, Universidade Federal do Rio de Janeiro, Rio de Janeiro, Brazil; 8 Intensive Care Unit and Postgraduate Program, A.C. Camargo Cancer Center, São Paulo, Brazil; 9 Research Institute, HCor-Hospital do Coração, São Paulo, Brazil; 10 Department of Intensive Care & Laboratory of Experimental Intensive Care and Anesthesiology (L·E·I·C·A), Academic Medical Center, Amsterdam, The Netherlands; 11 Mahidol–Oxford Tropical Medicine Research Unit (MORU), Faculty of Tropical Medicine, Mahidol University, Bangkok, Thailand

**Keywords:** Intensive care unit, Invasive ventilation, lung protection, Tidal volume, low tidal volume ventilation, Organizational factors

## Abstract

**Background:**

Survival benefit from low tidal volume (*V*_T_) ventilation (LTVV) has been demonstrated for patients with acute respiratory distress syndrome (ARDS), and patients not having ARDS could also benefit from this strategy. Organizational factors may play a role on adherence to LTVV. The present study aimed to identify organizational factors with an independent association with adherence to LTVV.

**Methods:**

Secondary analysis of the database of a multicenter two-phase study (prospective cohort followed by a cluster-randomized trial) performed in 118 Brazilian intensive care units. Patients under mechanical ventilation at day 2 were included. LTVV was defined as a *V*_T_ ≤ 8 ml/kg PBW on the second day of ventilation. Data on the type and number of beds of the hospital, teaching status, nursing, respiratory therapists and physician staffing, use of structured checklist, and presence of protocols were tested. A multivariable mixed-effect model was used to assess the association between organizational factors and adherence to LTVV.

**Results:**

The study included 5719 patients; 3340 (58%) patients received LTVV. A greater number of hospital beds (absolute difference 7.43% [95% confidence interval 0.61–14.24%]; *p *= 0.038), use of structured checklist during multidisciplinary rounds (5.10% [0.55–9.81%]; *p *= 0.030), and presence of at least one nurse per 10 patients during all shifts (17.24% [0.85–33.60%]; *p *= 0.045) were the only three factors that had an independent association with adherence to LTVV.

**Conclusions:**

Number of hospital beds, use of a structured checklist during multidisciplinary rounds, and nurse staffing are organizational factors associated with adherence to LTVV. These findings shed light on organizational factors that may improve ventilation in critically ill patients.

## Introduction

Survival benefit from low tidal volume (*V*_T_) ventilation (LTVV) has clearly been demonstrated for patients with acute respiratory distress syndrome (ARDS) [1]. Patients not having ARDS could also benefit from this strategy [2–5], albeit that tidal volumes may not always need to be as low as in patients with ARDS [6]. As it could be difficult to discriminate patients with ARDS from those not having ARDS [7, 8], one reasonable and pragmatic approach could be to use LTVV (defined as ventilation using a *V*_T_ ≤ 8 ml/kg predicted body weight [PBW]) in all invasively ventilated critically ill patients [9]. This is, at least, in part in line with international guidelines recommending a *V*_T_ of 4 to 8 ml/kg PBW in patients with ARDS [10], and with the suggestion not to use a *V*_T_ > 8 ml/kg PBW in patients not having ARDS [11–13].

Several studies have demonstrated an increased adherence to LTVV over recent decades [14–17]. Yet, many patients remain to receive ventilation with a too large *V*_T_ [7, 18, 19]. For instance, the ‘Large observational study to UNderstand the Global impact of Severe Acute respiratory FailurE’ (LUNG SAFE) study showed that as many as one-third of ARDS patients receive a *V*_T_ > 8 ml/kg PBW [7]. A similar proportion of patients without ARDS did not receive LTVV in the ‘PRatice of VENTilation in critically ill patients without ARDS’ (PRoVENT) study [18]. In the ‘Checklist During Multidisciplinary Visits for Reduction of Mortality in Intensive Care Units’ (CHECKLIST-ICU) study, even if the intervention led to a better adherence to LTVV as compared with the control group, only two-thirds of patients under invasive ventilation in the intervention group received a *V*_T_ ≤ 8 ml/kg PBW [19].

Organizational factors associated with a better outcome in ICU patients include intensity of doctor, nurse and respiratory therapist staffing [20–24], continuity of care [25, 26], and use of multidisciplinary rounds and structured handovers [27, 28]. Yet, no study evaluated which organizational factors are related to adherence to LTVV. Therefore, the CHECKLIST-ICU database was used to identify organizational factors that are independently associated with adherence to LTVV.

## Methods

### Study design and patients

The study protocol of the CHECKLIST-ICU study was prepublished [29] and registered (clinicaltrials.gov, study identifier NCT01785966), and the results of the primary analysis were reported recently [19]. In brief, the CHECKLIST-ICU study consisted of two phases. In phase I, organizational factors and clinical outcomes were collected in 118 Brazilian adult ICUs, from August 2013 to March 2014. In phase II, the ICUs were randomized to a quality improvement intervention or to usual care, from April 2014 to November 2014. The quality improvement intervention consisted of a checklist and discussion of goals of care during daily multidisciplinary rounds, followed by clinician prompting to ensure checklist adherence and goals of care.

The checklist assessed prevention and management of lung injury, venous thromboembolism, ventilator-associated pneumonia, central line-associated bloodstream and urinary tract infections, nutritional targets, analgesia and sedation goals, detection of sepsis and ARDS, and antibiotic initiation and stewardship. In specific, for prevention of lung injury, it was advised to adhere to LTVV and to assess readiness for extubation. Other care processes, with the exception of the checklist, discussion of goals of care and clinician prompting were unchanged between the two phases of the study. The institutional review boards of all centers approved the study. The funding source had no role in the analysis or publication decisions.

The CHECKLIST-ICU restricted participation to patients 18 years or older; and with an ICU stay longer than 48 h. Patients in whom a high probability of early death was anticipated (defined as death occurring between the 48th and 72nd hours of the ICU stay), patients receiving exclusive palliative care, as well as patients who were suspected of or had a diagnosis of brain death were excluded. The current analysis uses data from both study phases, but restricted to patients who had received invasive ventilation for at least 2 days. This period was chosen for two reasons: first, to guarantee sufficient time to evaluate exposure to the intervention, and second, because the majority of patients were extubated within 3 days.

### Data collection and definitions

The database of the CHECKLIST-ICU study contains baseline information [age, gender, reason for admission, type of admission (clinical, elective or urgent surgery), illness severity (Simplified Acute Physiology Score [SAPS] 3 and Sequential Organ Failure Assessment [SOFA])], study phase (phase I or II), ICU and hospital mortality, and ICU and hospital length of stay.

For ventilation evaluation, in the original trial *V*_T_ size was recorded on day 2, 5, 8, 11, 14 and 17. The target *V*_T_ in the checklist was ≤ 8 ml/kg PBW in all days, with PBW calculated as 50 + 0.91*(height [in cm] − 152.4) for males and 45.5 + 0.91*(height [in cm] − 152.4) for females. For the purpose of the current analysis, adherence to LTVV was defined as *V*_T_ of ≤ 8 ml/kg PBW at the second day of ventilation only.

In the participating units, physiotherapists were responsible for the ventilator settings and the physical therapy. However, ventilatory management was adjusted according to decisions taken in multidisciplinary rounds, and after approval of the physician in charge. In some units, in particular when a physiotherapist was not a member of the team, nurses were allowed to change ventilatory settings.

The following organizational data related to the process of care of each participating ICU were collected in the CHECKLIST-ICU study and considered in the present study:

- Level of hospital (tertiary vs non-tertiary);
- Type of hospital (specialty vs general);
- Teaching status (university affiliated vs not university affiliated);
- Number of hospital beds (according to tertiles in the present study);
- Number of ICU beds (according to tertiles in the present study);
- Presence of multidisciplinary rounds (yes vs no);
- Use of a structured checklist during rounds (yes vs no);
- Presence of sedation and analgesia, and weaning, and prevention of ventilator-associated pneumonia (VAP) protocols (yes vs no);
- Presence of board-certified ICU consultant (morning and afternoon vs morning or afternoon);
- Presence of at least one physician for each 10 patients in all shifts; (yes vs no);
- Presence of one board-certified respiratory therapist coordinator (yes vs no);
- Presence of one respiratory therapist for each 10 patients in all shifts (yes vs no);
- Presence of at least one nurse per 10 patients during all shifts (yes vs no).

### Outcomes

The primary outcome of this analysis was the organizational factors associated with adherence to LTVV. Secondary outcomes were the adherence to LTVV on the second day of ventilation, and the impact of adherence to LTVV in ICU and hospital mortality.

### Statistical analyses

Continuous variables are presented as mean ± standard deviation and compared with Student’s *t* test. Categorical variables are presented as absolute numbers and proportions and compared with the Chi square test or Fisher’s exact test, where appropriate. All analyses were performed using multilevel (patients nested in study centers, within the phases of the trial) mixed modeling with centers as random effect and the phase of the study as a fixed effect. Absolute difference between the groups with the respective 95%-confidence interval (95% CI) was calculated from a mixed-effect linear model and reported as mean differences for continuous variables and as risk differences for categorical variables.

The association between organizational factors and use of LTVV was assessed with a mixed-effect generalized linear model and the variables included in the multivariable models were defined according to clinical rationale and when a *p* value < 0.20 was found in the univariable models. The final multivariable model was adjusted for patient severity, according to SAPS 3 and SOFA. Heterogeneity between the phases of the trial was determined by fitting a fixed interaction term between the phase and organizational factors associated with use of LTVV, while overall effect is reported with phase treated as a fixed effect and centers treated as a random effect. The use of a structured checklist was not considered in the heterogeneity analysis, since it was used only in one of the phases of the study, i.e., in the cluster-randomized trial.

The impact of LTVV on ICU and hospital mortality was also assessed with a mixed-effect generalized linear model considering a binomial distribution, adjusted for SAPS 3 and SOFA at the patient level.

Continuous variables were standardized before being included in the multivariable models described above to improve convergence. All analyses were performed using the R (R, version 3.6.0, Core Team, Vienna, Austria, 2016) software, and a two-sided alpha level of 0.05 was considered.

## Results

**Fig. 1:**
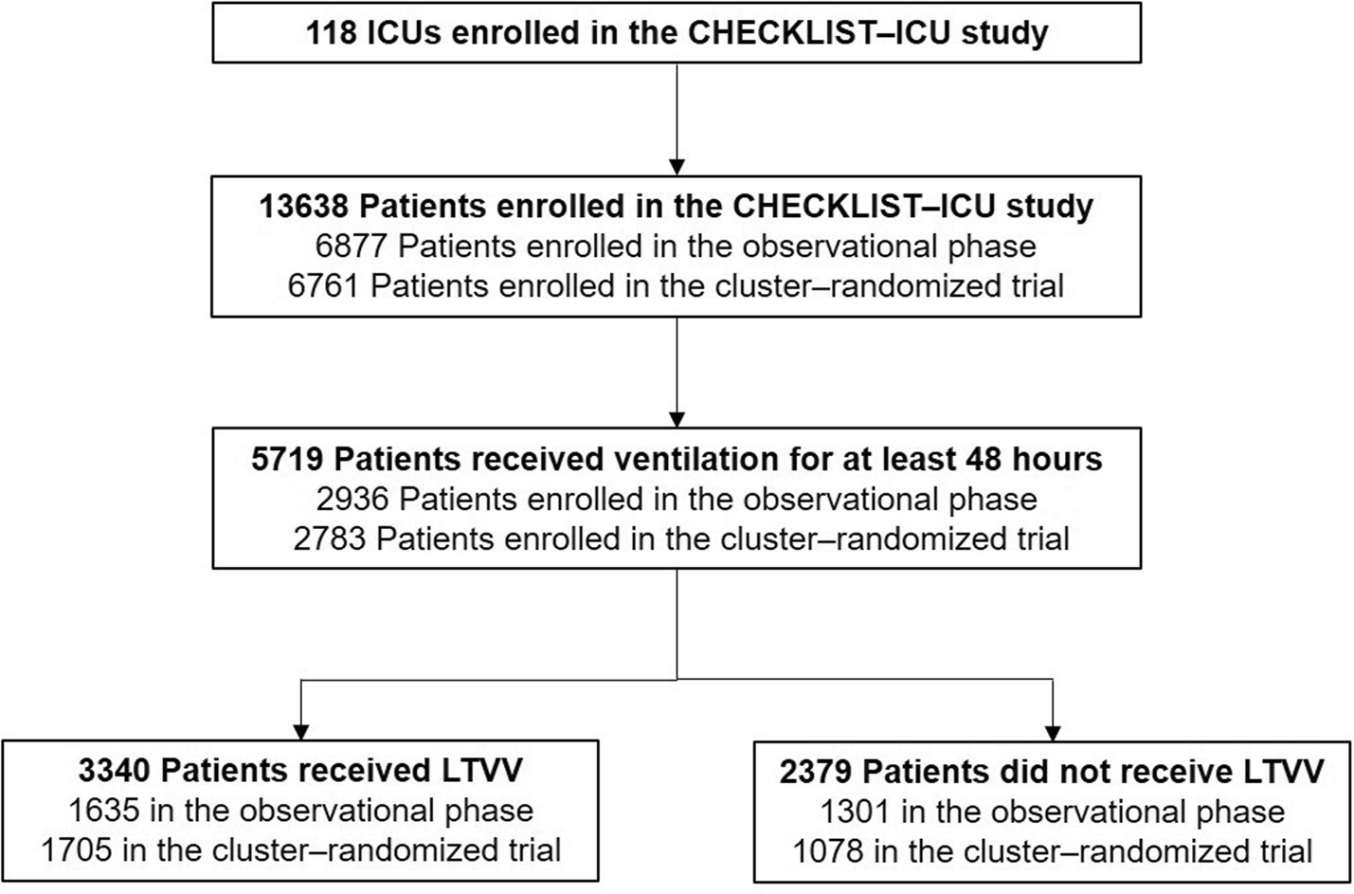
Patient flowchart. LTVV, low tidal volume ventilation

The database of the CHECKLIST-ICU study included data of a total 13,638 patients in 118 ICUs. Of them, 5719 patients had received invasive ventilation for at least 2 days, 2936 (51.3%) in phase I, and 2783 (48.7%) in phase II (Fig. 1). Baseline characteristics of patients who received LTVV (3340 patients, 58% of all patients) and patients who did not receive LTTV at the second day of ventilation (2379 patients, 42% of all patients) are shown in Table 1. Most admissions were medical, and the main reasons for admission were sepsis and acute respiratory failure. *V*_T_ data were available for all patients on day 2.

**Table 1:** Baseline characteristics of the included patients

	All patients (*n* = 5719)	LTVV* (*n* = 3340)	No LTVV* (*n* = 2379)	*p* value
Age, years	63.0 ± 19.2	60.9 ± 19.5	66.0 ± 18.4	< 0.001
Female gender	2441 (42.7)	1022 (30.6)	1419 (59.6)	< 0.001
Predicted body weight, kg	60.5 ± 10.1	63.9 ± 9.2	55.7 ± 9.2	< 0.001
Height, cm	166 ± 9	169 ± 9	162 ± 9	< 0.001
SAPS III	62.3 ± 17.1	61.7 ± 17.4	63.2 ± 16.7	0.002
SOFA	6.6 ± 3.7	6.5 ± 3.7	6.7 ± 3.7	0.068
Study phase				< 0.001
Observational	2936 (51.3)	1635 (49.0)	1301 (54.7)	
Interventional	2783 (48.7)	1705 (51.0)	1078 (45.3)	
Type of admission				< 0.001
Medical	4326 (75.6)	2486 (74.4)	1840 (77.3)	
Elective surgery	371 (6.5)	202 (6.0)	169 (7.1)	
Urgent surgery	1022 (17.9)	652 (19.5)	370 (15.6)	
Reason for ICU admission				0.078
Postoperative care	694 (12.1)	419 (12.5)	275 (11.6)	
Acute respiratory failure	1323 (23.1)	752 (22.5)	571 (24.0)	
Cardiac arrest	174 (3.0)	100 (3.0)	74 (3.1)	
Neurological disorders	816 (14.3)	472 (14.1)	344 (14.5)	
Liver disorders	74 (1.3)	44 (1.3)	30 (1.3)	
Gastrointestinal disorders	105 (1.8)	59 (1.8)	46 (1.9)	
Sepsis	1063 (18.6)	601 (18.0)	462 (19.4)	
Shock (not considering sepsis)	98 (1.7)	64 (1.9)	34 (1.4)	
Cardiovascular disorders	237 (4.1)	135 (4.0)	102 (4.3)	
Kidney disorders	188 (3.3)	106 (3.2)	82 (3.4)	
Hematological disorders	45 (0.8)	20 (0.6)	25 (1.1)	
Others	902 (15.8)	568 (17.0)	334 (14.0)	
Co-morbidities				
Cancer	444 (7.8)	233 (7.0)	211 (8.9)	0.010
Heart failure	342 (6.0)	194 (5.8)	148 (6.2)	0.554
Cirrhosis	153 (2.7)	98 (2.9)	55 (2.3)	0.176
AIDS	265 (4.6)	177 (5.3)	88 (3.7)	0.006

Data are mean ± standard deviation or *N* (%)

*SAPS* Simplified Acute Physiology Score, *SOFA* Sequential Organ Failure Assessment, *ICU* intensive care unit; *AIDS* acquired immune deficiency syndrome

* LTVV defined in tidal volume ≤ 8 ml/kg PBW in the second day of ventilation

**Fig. 2:**
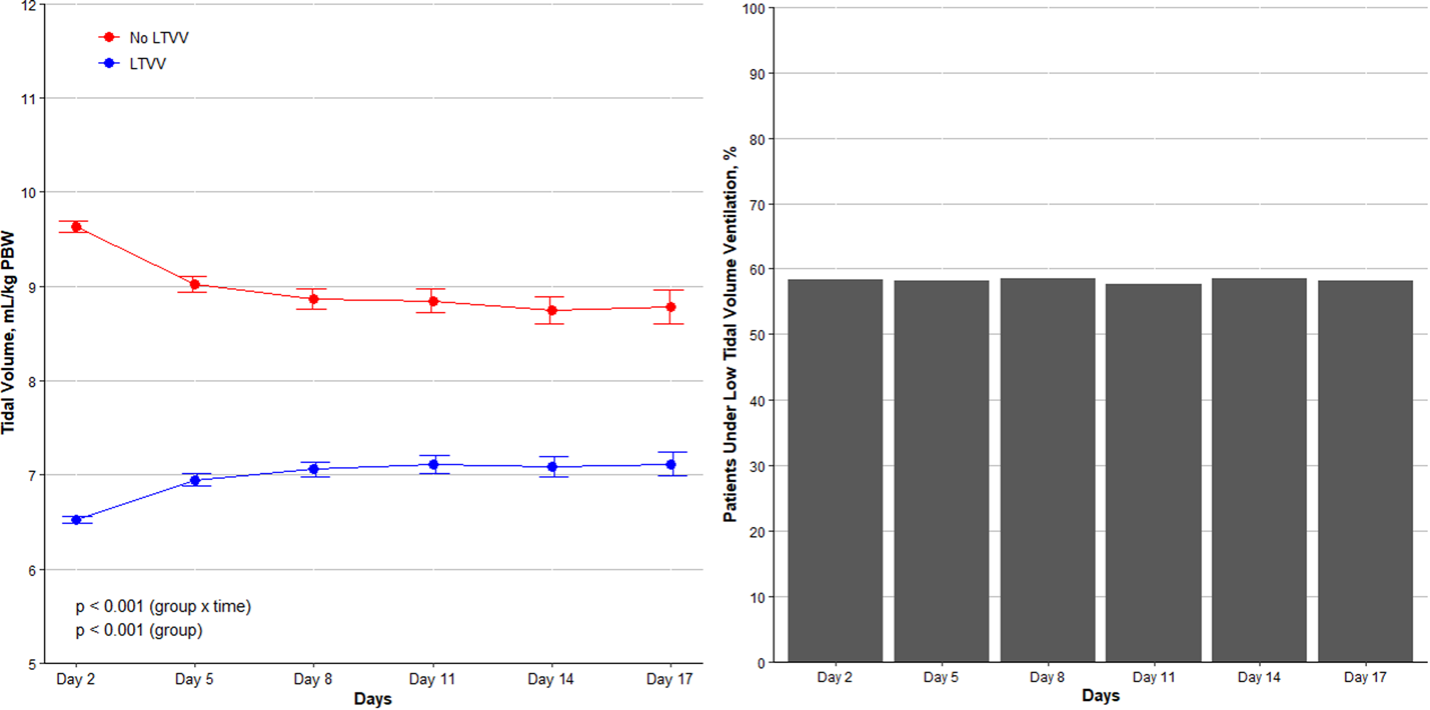
Tidal volume and frequency of the use of low tidal volume ventilation over the first 17 days of follow-up. Circles are the mean and error bars the 95% confidence interval. Unadjusted mixed-effect longitudinal models with random intercept for patients and center, and with phase of the study, group, days and the interaction of group × days as fixed effects. *p* values for the group reflect the overall test for difference between groups across the follow-up while *p* values for the group × days interaction evaluate if change over time differed by group

The proportion of patients receiving LTVV was 58.4% (95% CI, 57.1% to 59.7%) on day 2, and did not change thereafter (Fig. 2). Patients who received LTVV were younger, taller, were more often male, and were less severely ill according to their SAPS and SOFA scores.

Organizational factors with an association with adherence to LTVV in the unadjusted analysis are shown in Table 2. In the adjusted analysis, number of hospital beds (absolute difference 7.43 [0.61 to 14.24]; *p* = 0.038), use of a structured checklist during multidisciplinary rounds (absolute difference 5.10 [0.55 to 9.81]; *p* = 0.030), and presence of at least one nurse per 10 patients during all shifts (absolute difference 17.24 [0.85 to 33.60]; *p* = 0.045) were associated with adherence to LTVV (Table 3).

**Table 2:** Factor associated with the use of low tidal volume

	All patients (*n* = 5719)	LTVV* (*n* = 3340)	No LTVV* (*n* = 2379)	Absolute difference** (95% CI)	*p* value
Related to the trial					
Use of checklist	1279 (22.4)	843 (25.2)	436 (18.3)	4.41 (− 0.20 to 9.02)	0.061
Related to the hospital					
Tertiary	4605 (81.5)	2742 (83.2)	1863 (79.1)	5.04 (− 2.29 to 12.35)	0.180
Specialty	1060 (18.8)	691 (21.0)	369 (15.7)	5.61 (− 2.89 to 14.06)	0.197
University	2202 (39.0)	1309 (39.7)	893 (37.9)	− 2.23 (− 8.57 to 4.12)	0.493
Number of beds					
< 157	1887 (33.4)	1086 (32.9)	801 (34.0)	8.62 (2.08 to 15.17)^a^	0.011
157–324	1906 (33.7)	1041 (31.6)	865 (36.7)		
> 324	1857 (32.9)	1169 (35.5)	688 (29.2)		
Number of ICU beds					0.445
< 11	2362 (41.8)	1364 (41.4)	998 (42.4)	1.85 (− 5.17 to 8.87)^b^	0.606
11–21	1774 (31.4)	1028 (31.2)	746 (31.7)		
> 21	1514 (26.8)	904 (27.4)	610 (25.9)		
Related to organization					
Multidisciplinary rounds	3444 (60.2)	2068 (61.9)	1376 (57.8)	− 0.15 (− 3.63 to 3.34)	0.932
Sedation protocol	2620 (46.4)	1509 (45.8)	1111 (47.2)	− 2.02 (− 8.08 to 4.02)	0.514
Analgesia protocol	2285 (40.4)	1342 (40.7)	943 (40.1)	2.18 (− 3.99 to 8.37)	0.490
Weaning protocol	3644 (64.5)	2090 (63.4)	1554 (66.0)	− 1.36 (− 7.73 to 5.02)	0.677
VAP protocol	3621 (64.1)	2046 (62.1)	1575 (66.9)	− 3.58 (− 9.89 to 2.74)	0.269
Board-certified consultant					
None	371 (6.6)	204 (6.2)	167 (7.1)	4.11 (− 1.92 to 10.15)^c^	0.185
Full-time	3015 (53.4)	1821 (55.2)	1194 (50.7)		
Half-time	2264 (40.1)	1271 (38.6)	993 (42.2)		
1:10 physician	5466 (96.7)	3205 (97.2)	2261 (96.0)	6.74 (− 8.75 to 22.22)	0.395
Board-certified RT coordinator	3920 (69.4)	2309 (70.1)	1611 (68.4)	− 2.69 (− 9.41 to 4.06)	0.436
1:10 respiratory therapist					
None	108 (1.9)	69 (2.1)	39 (1.7)	− 2.53 (− 8.59 to 3.53)^c^	0.415
Full-time	2438 (43.2)	1383 (42.0)	1055 (44.8)		
Half-time	3104 (54.9)	1844 (55.9)	1260 (53.5)		
1:10 nurse	5482 (97.0)	3227 (97.9)	2255 (95.8)	20.24 (3.75 to 36.76)	0.018

*VAP* ventilator-associated pneumonia, *RT* respiratory therapist

* LTVV defined in tidal volume ≤ 8 ml/kg PBW in the second day of ventilation

** Calculated as the risk difference from a mixed-effect model with the phase of the study as a fixed effect and the hospital as random effect. The comparison is the difference in the use of low tidal volume ventilation among each factor

^a^Comparison of > 324 vs ≤ 324 beds

^b^Comparison of > 21 vs ≤ 21 beds

^c^Comparison of full-time vs. none/half-time

**Table 3:** Organizational factors associated with the use of low tidal volume ventilation

	Absolute difference* (95% confidence interval)	*p* value
Adjustment by severity of illness		
SAPS III	− 2.64 (− 4.27 to − 1.00)	0.001
SOFA	1.28 (− 0.40 to 2.92)	0.131
Trial related		
Use of structured checklist	5.10 (0.55 to 9.81)	0.030
Hospital related		
Tertiary hospital	0.10 (− 7.44 to 7.66)	0.978
Specialty hospital	4.34 (− 3.76 to 12.49)	0.306
Number of hospital beds > 324	7.43 (0.61 to 14.24)	0.038
Organizational factors		
Board-certified consultant	2.55 (− 3.33 to 8.44)	0.406
At least one nurse per 10 patients during all shifts	17.24 (0.85 to 33.60)	0.045

*SAPS* Simplified Acute Physiology Score, *SOFA* Sequential Organ Failure Assessment

Mixed-effect generalized linear model considering the phase of the study and the variables as fixed effect and the center as random effect. Continuous variables were standardized before inclusion in the model

* Higher positive values indicate higher adherence to low tidal volume ventilation

The effect of the phase of the study was not significant (2.34 [95% confidence interval − 0.96 to 5.87]; *p* = 0.161)

The interaction between the phase of the study and the number of hospital beds was not significant (*p* = 0.254)

The interaction between the phase of the study and the presence of one nurse for every 10 patients in all shifts was significant (*p* = 0.032)

There was no heterogeneity between study phases and the effect of number of hospital beds on adherence to LTVV (*p* = 0.254). There was an interaction between study phases and the effect of the presence of at least one nurse per 10 patients during all shifts on adherence to LTVV (estimate, − 16.13 [− 31.0 to − 1.41]; *p* = 0.032). In the second phase of the study, presence of at least one nurse per 10 patients during all shifts and adherence to LTVV were no longer associated (Additional file 1: Figure S1).

There was no difference in ICU (absolute difference, 0.27 [− 2.29 to 2.84]; *p* = 0.833) or hospital mortality (absolute difference, 0.03 [− 2.51 to 2.57]; *p* = 0.980) according to the use LTVV (Additional file 1: Table S1).

## Discussion

This study suggests that the larger centers with a higher number of hospital beds, use of structured checklists during multidisciplinary rounds, and the presence of at least one nurse for every 10 patients in all shifts are the three organizational factors associated with adherence to LTVV. Interestingly, *V*_T_ stays remarkable unchanged over the total duration of ventilation, i.e., *V*_T_ does not change beyond the second day of ventilation. Mortality was not different between patients who received and those who did not receive LTVV.

The finding that the number of hospital beds is associated with a higher adherence to LTVV might be explained as follows. First, ICUs in larger hospitals usually have higher number of caregivers, more experienced teams, and also higher volumes of invasively ventilated patients. Second, ICU bed supply and quality of care in ICUs are usually related to the size of a hospital [30, 31], and smaller hospitals frequently suffer with lack of beds and consequently inappropriate allocation, which is also associated with a lower quality of care [32]. Nevertheless, in Brazil usually the larger hospitals are the public university-affiliated hospitals, and in the present analyses this intersection could not be completely assessed. However, the association among larger hospitals and adherence to LTVV could reflect, indirectly, a higher adherence in teaching hospitals also. All those characteristics, however, were not directly collected and measured in the CHECKLIST-ICU study.

The finding that the use of a structured checklist during multidisciplinary rounds is associated with adherence to LTVV is in line with findings of previous studies. One study in invasively ventilated ICU patients with ARDS showed that use of a written ventilation protocol was associated with higher use of LTVV [33]. Another study in critically ill ICU patients demonstrated that charts installed on ventilators showing the adequate *V*_T_ target (4–6 ml/kg PBW) for each patient led to a significant decrease in *V*_T_ [24]. Such findings suggest that adoption of written documents such as protocols, checklists and charts may improve adherence to LTVV and ensure translation of evidence for benefit, in this case of LTVV, into clinical practice. Of note, protocols for mechanical ventilation often do not incorporate LTVV strategies [34], and use of a protocol does not guarantee that all patients will receive the best practice [35]. However, one large Brazilian study demonstrated that the implementation of protocols was associated with better patient outcomes and more efficient use of resources [27].

The finding that adherence to LTVV increased with presence of more nurses is also in line with previous findings [20–22]. A nurse-to-patient ratio  > 1:1.5 was independently associated with a lower risk of in-hospital death in a large multicenter cohort of ICU patients in a worldwide study [22]. Vice versa, a lower ICU nurse staffing was associated with adverse outcomes in a study in hospitals across the USA [21]. In Brazil, ICU nurse staffing varies considerably depending on local regulations. However, a study in 93 Brazilian ICUs showed that ICUs where nurses have higher autonomy, including start of weaning from ventilation and the titrating FiO_2_, have better outcomes compared to ICUs where there is less nurse autonomy [36]. After implementation of the structured checklist in the second phase of the CHECKLIST-ICU study, the positive effect of one nurse for every 10 patients in all shifts on the use of LTVV was no longer associated with compliance with LTVV. This finding is important, and suggests that a structured checklist can increase the adoption of LTVV, even in institutions where the nurse-to-patient ratio is low. At the end, the impact of a higher nurse-to-patient ratio on adherence to LTVV can be explained by several different factors, including (1) adoption of the checklist, per se; (2) better use of the checklist by healthcare providers like respiratory therapists, typically more present in ICUs with a higher nurse-to-patient ratio; and (3) presence of more nurses, who can adjust ventilator settings.

Use of LTVV was not associated with lower mortality as described in previous studies [3, 5, 37]. This may be explained by the small difference in *V*_T_ between the two groups, i.e., 6.7 (5.9–7.4) vs 9.2 (8.5–10.2) ml/kg PBW. This is different from previous studies that used *V*_T_ of as high as 12 ml/kg PBW for comparison. Of note, one recent randomized clinical trial in patients not having ARDS did found beneficial effects of a low *V*_T_ strategy [resulting in *V*_T_ of 6.6 (5.5–8.7) ml/kg PBW] when compared to an intermediate *V*_T_ strategy [resulting in *V*_T_ of 9.3 (8.1–10.1) ml/kg PBW] [6]. Despite this, identifying factors that have an effect of use of LTVV could help improving ICU organization as well as safety of invasively ventilated patients in general.

Interestingly, the current study suggests that early use of LTVV is associated with continued use of ventilation with a low *V*_T_ on successive days. This finding shows that patients who had *V*_T_ correctly titrated to PBW early on ICU admission were more likely to continue with LTVV. This finding could suggest that adherence to LTVV is not necessarily related to severity of diseases. Vice versa, a patient who starts ventilation with a too high *V*_T_ is at risk of not receiving LTVV a later time point.

The present study has limitations. First, it is a secondary analysis of a study designed to evaluate the effectiveness of a multifaceted quality improvement strategy including a checklist in ICU. Thus, some important data regarding mechanical ventilation and patient severity are missing, like other ventilatory parameters, and the development of complications. Second, all included ICUs are from Brazil, and it is widely known that organizational factors and process of care differ worldwide. For example, in the present study only half of the patients were admitted to ICUs with board-certified intensivists on the morning and afternoon shifts [27, 28]. Indeed, the lower number of board-certified intensivists is a well-known problem in low-income countries [38]. Third, no information about the presence of ARDS is available and this could have influenced the adoption of LTVV and the impact of LTVV on hospital mortality. Indeed, in the here studied cohort the majority of the patients were extubated within 3 days, thus the percentage of patients with ARDS was probably low, which can partly explain the lack of power to detect any association between LTVV and mortality. Fourth, it is important to emphasize that current findings may only be generalizable to settings with a comparable infrastructure, especially the nurse-to-patient ratio, since Brazil has a much lower nurse-to-patient ratio then ICUs in, e.g., Europe and the US [22]. Finally, no information about the ventilatory mode was available, and this could have influenced the results.

## Conclusions

In Brazil, the number of hospital beds, use of a structured checklist during multidisciplinary rounds, and presence of at least one nurse for every 10 patients in all shifts were the only organizational factors associated with adherence to LTVV. These findings shed light on organizational factors that may increase adherence to LTVV.

## Supplementary information

**Additional file 1.** Online Supplement.
