# Supplementary material for: Organizational factors associated with adherence to low tidal volume ventilation: a secondary analysis of the CHECKLIST-ICU database
Source: Ann Intensive Care. 2020 Jun 1;10:68. doi: 10.1186/s13613-020-00687-3 (PMC7266115; doi:10.1186/s13613-020-00687-3)
Supplement: Supplementary file 1 — Additional file 1. Online Supplement. [file 13613_2020_687_MOESM1_ESM.docx]

**ESM**

**Organizational Factors Associated with Adherence to Low Tidal Volume Ventilation – a posthoc analysis of the CHECKLIST–ICU**

| **Table S1 – Clinical outcomes of the patients according to use of low tidal volume ventilation** | | | | | | | |
| --- | --- | --- | --- | --- | --- | --- | --- |
|  | **All Patients**  **(*n* = 5719)** | **LTVV***  **(*n* = 3340)** | **No LTVV***  **(*n* = 2379)** | **Unadjusted Analysis**** | | **Adjusted Analysis***** | |
|  |  |  |  | **Absolute Difference**  **(95% CI)** | ***p* value** | **Absolute Difference**  **(95% CI)** | ***p* value** |
| ICU mortality | 2583 (45.2) | 1486 (44.5) | 1097 (46.1) | -0.5 (-3.2 to 2.1) | 0.690 | 0.3 (-2.3 to 2.8) | 0.833 |
| Hospital mortality | 3068 (53.7) | 1761 (52.7) | 1307 (55.0) | -1.0 ( -3.6 to 1.7) | 0.478 | 0.0 (-2.5 to 2.6) | 0.980 |
| * LTVV defined in tidal volume ≤ 8 ml/kg PBW in the second day of ventilation  ** calculated as the risk difference from a mixed–effect model with the phase of the study as a fixed effect and the hospital as random effect.  *** further adjusted by SAPS II and SOFA | | | | | | | |

**Figure S1 – Interaction among phase of the study and the presence of presence of one nurse for every 10 patients**

**
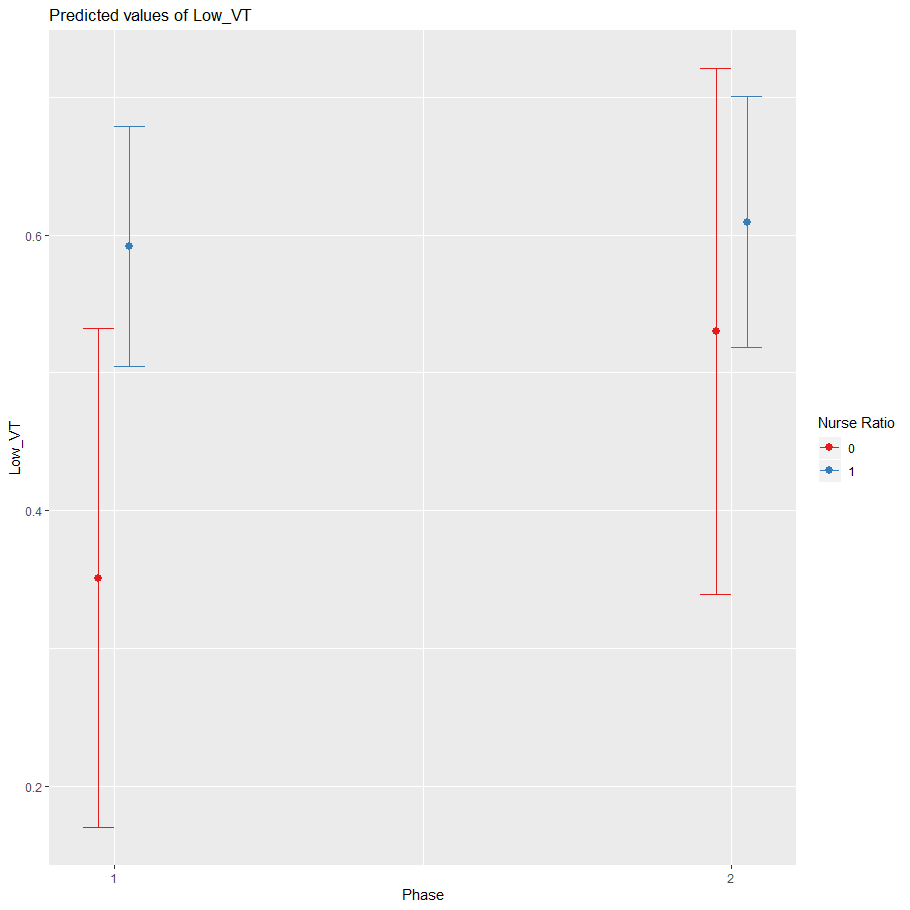
**

*p* for interaction among phase of the study and the presence of one nurse for every 10 patients (0.032). Values derived from the full multivariable model (Figure 2)
